# Non-Verbal Working Memory in Post-Stroke Motor Aphasia: A Pilot Study Using the Tactual Span

**DOI:** 10.3390/neurolint17040058

**Published:** 2025-04-17

**Authors:** Eyal Heled, Ohad Levi, Elana Strobinsky, Gabi Zeilig

**Affiliations:** 1 Department of Psychology, Ariel University, Ariel 40700, Israel; levi1ohad1i@gmail.com (O.L.); strobelana@gmail.com (E.S.); 2 The Rehabilitation Center, Sheba Medical Center, Ramat-Gan 5265601, Israel; gabi.zeilig@sheba.health.gov.il; 3 Faculty of Health Professionals, Ono Academic College, Kiryat-Ono 5545001, Israel

**Keywords:** working memory, Tactual Span, span task, stroke, aphasia

## Abstract

**Background**: Working memory (WM) impairment is a potential consequence of motor aphasia resulting from left-hemisphere ischemic stroke. While verbal WM has been studied extensively in this disorder, research regarding non-verbal modalities remains limited, particularly visuospatial WM, tactile WM, and the relationship between them. Additionally, language impairments limit the ability to assess WM in aphasia patients, highlighting the necessity of non-verbal diagnostic tools in clinical practice. The current study’s objectives were to compare tactile and visuospatial WM in patients with post-stroke motor aphasia and to validate the one-hand version of the Tactual Span task as a clinical measure of WM. **Methods**: A total of 29 participants—14 with post-stroke motor aphasia and 15 healthy controls—completed a battery of cognitive tests, including the Raven’s Colored Progressive Matrices Test, the Visuospatial Span, the Tactual Span, and a visual 1-Back task. **Results**: There was significantly lower performance across all WM tasks in the aphasia group compared to the controls. Additionally, the Tactual Span successfully discriminated between patients and controls, showing sensitivity estimates of 92.9% and a specificity of 66.7%, with a cut-off score of 4.5 (AUC = 0.91), for the forward stage. The backward stage revealed a sensitivity of 71.4% and a specificity of 73.3%, with a cut-off score of 3.5 (AUC = 0.83). **Conclusions**: The findings may suggest that non-verbal WM impairment in post-stroke aphasia affects both visuospatial and tactile modalities similarly. Furthermore, the Tactual Span appears to be sensitive to left-hemisphere stroke damage, suggesting its potential utility as a clinical tool for WM assessment in patients with motor aphasia.

## 1. Introduction

A left-hemisphere ischemic stroke may result in motor aphasia, which impairs skills such as oral fluency, comprehension, repetition, naming, and reading [1,2,3]. Although aphasia is predominantly known for its effects on speech, studies reveal that these effects are not limited to language production, processing, or linguistic comprehension. Rather, aphasia results in a complex interplay of impairments across various cognitive functions, encompassing both verbal and non-verbal domains [4,5,6,7]. Executive functioning, attention, logical reasoning, visuospatial perception, and orientation are a few examples of such impaired abilities [8,9,10,11]. These abilities are associated with semantic cognitive deficits and other language-related challenges, significantly influencing the recovery and rehabilitation process in individuals with aphasia [3].

Another complex neurocognitive ability shown to be impacted by stroke is working memory (WM), which is defined as the ability to temporarily store information in an active, accessible state and manipulate it to achieve specific goals [11,12,13,14]. WM is critically essential for various cognitive abilities in daily life, such as learning, attention, and language comprehension [12,15,16], as well as in the functional, social, and occupational domains [17,18]. Additionally, deficits in WM can adversely impact several language processes in individuals with aphasia, such as lexical–semantic processing and communication abilities [19,20].

Verbal WM impairments in stroke-induced aphasia patients have been linked to lesions in phonologically related regions such as the left ventral premotor and somatosensory cortex, supramarginal gyrus, left dorsal temporoparietal system, and left planum temporale [21,22,23,24,25]. However, other studies have suggested that lesions to parts of the left hemisphere may also cause problems with visuospatial WM [26,27,28,29,30]. A functional magnetic resonance imaging review on non-verbal WM functions has highlighted the involvement of the left medial frontal cortex, left superior parietal lobule, and left precuneus [31].

Paulraj et al. [32] examined the effect of the left hemisphere on visuospatial WM by assessing spatial span task performance in 50 stroke patients with left-hemisphere damage. Their findings revealed that some participants demonstrated significant spatial WM deficits, with 28% showing poor performance on forward spatial span tasks and 16% on backward spatial span tasks. Notably, these impairments were independent of language deficits, highlighting the role of a left-hemisphere fronto-parietal network in spatial WM, and indicating that left, and not just right, brain areas are also associated with visuospatial processing.

Several explanations have been proposed to explain why left-hemisphere stroke affects both visuospatial WM in addition to verbal WM, with two appearing most prominent. The first explanation argues that the ability to comprehend and follow verbal instructions is damaged, thus affecting performance in non-verbal tasks [1,32]. Secondly, left-hemisphere stroke damages neural networks responsible for processing and storing non-verbal WM stimuli [33].

Clinicians often encounter difficulties when assessing cognitive abilities in aphasia patients, because many cognitive tests rely on verbal functioning, and non-verbal tests are not well validated for this population [1,17,34,35,36]. Fonseca et al. [37] added that impaired communication abilities can disrupt standard cognitive assessments, reducing the reliability of tools validated on healthy populations, as they have not been modified for use with aphasia patients. Such assessment is important because it helps to design the patients’ treatment program within rehabilitation settings [38]. Consequently, non-verbal tests validated for aphasia patients could be valuable for assessing WM.

Examples of such tests are specific non-verbal variations of the span task, such as the spatial span [39]. These are commonly used tools for assessing WM [40], particularly in patients with aphasia [11]. In these tasks, participants are presented with sequences from various modalities, requiring them to recall items in both precise (forward) and reverse (backward) orders over multiple trials. According to Snyder et al. [41], the forward recall stage mainly measures storage capacity, whereas the backward recall stage assesses manipulation skills.

Indeed, the verbal and visuospatial modalities are used in clinical cognitive assessment far more often than the tactile modality. Murray et al. [11] conducted a review to identify standardized verbal and non-verbal WM measures for individuals with aphasia. However, their search did not include the tactile modality, indicating that this is not considered a potential domain for assessing WM, and that tactile WM has not yet been tested on aphasia patients despite its potential to provide insight into WM function [42,43].

To the best of our knowledge, no studies have specifically examined tactile WM either alone or compared to visuospatial WM in aphasia patients. Tactile WM involves temporarily retaining and manipulating tactile information [44], and is integral to various neurocognitive processes, including memory, perception, and attention [45]. This research gap is particularly notable considering Baddeley’s [12] theoretical assumption that tactile WM storage is incorporated within the visuospatial short-term compartment. Further evidence supports this notion, as it highlights the shared neural architecture underlying both memory systems. For example, Schmidt and Blankenburg [46] demonstrated that both the left and right posterior parietal cortices, as well as premotor regions, retain information during the delay phase of tactile and visuospatial WM tasks. This is consistent with other studies that have also revealed prefrontal cortex activation in both modalities [29,32,47,48,49]. Such findings suggest that damage to these areas in the left hemisphere could impair both tactile and visuospatial WM. Consequently, investigating this relationship should provide insightful empirical data and possible clinical significance.

Recently, the Tactual Span task was developed as a novel tool for assessing WM in the tactile modality using both hands, demonstrating validity and reliability [50,51]. Research applying the Tactual Span indicates that tactile WM capacity is generally lower compared to auditory and visuospatial span tasks in both young adults and elderly populations [43,50], potentially attributable to the limited engagement with fine tactile discrimination in daily activities [52]. However, despite being more cognitively demanding, tactile WM exhibits developmental plasticity in children and shows greater potential for improvement compared to other sensory modalities [53].

Moreover, the Tactual Span task has been validated for use in individuals with blindness [54], and further investigation has revealed that blindness demonstrates enhanced tactile WM performance compared to those with hearing impairments and neurotypical controls [42]. This disparity is attributed to the everyday engagement with tactile information (Braille reading) among individuals with blindness. Conversely, individuals with deafness show no significant advantage in tactile WM over the control group, likely due to equivalent tactile modality usage [55]. Expanding the task’s utility, a one-handed version has recently been developed and found to be effective in evaluating WM [56]. Such adaptation potentially extends the task’s applicability, making it suitable for individuals with hemiparesis or unilateral motor limitations.

Therefore, the current study aimed to validate the Tactual Span for use in post-stroke aphasia patients by comparing WM performance across visuospatial and tactile modalities among stroke patients with motor aphasia using the Tactual Span and other well-established visuo-spatial WM tasks [39,57]. An additional means to reach that aim was to evaluate whether the one-hand version of the Tactual Span effectively discriminates between patients and controls. We hypothesized that post-stroke patients would perform worse than controls across all WM tasks, with the Tactual Span likely being the most discriminative measure in both short-term and WM assessments. This expectation was based on the premise that the tactile modality is utilized less frequently in everyday life [52], 2005), potentially making tactile tasks more challenging and thus more sensitive to cognitive impairments. The findings may provide insights into how left-hemisphere stroke damage affects tactile WM specifically, and non-verbal WM in general, and how it relates to aphasia. Furthermore, as there is a notable scarcity of validated instruments necessary for post-stroke rehabilitation, it may improve the clinical toolkit for clinicians working with motor aphasia patients [3,11,34,58].

## 2. Method

### 2.1. Participants

The statistical power analysis software G*Power (version 3.1.9.7; [59,60]) was utilized to determine the appropriate sample size for this study. Based on research literature, which reports a significant decline in higher cognitive functions, particularly in WM, among individuals with post-stroke aphasia, an expected effect size of 0.6 was selected [18,20,61,62,63]. The calculation was conducted using a standard statistical power of 0.80 and a significance level of 0.05, indicating that a minimum of twenty-eight participants would be required to achieve adequate statistical power. Participants with post-stroke aphasia were recruited from the neurological rehabilitation department at Sheba Medical Center and via referrals from private practice speech therapists. Healthy controls were sourced from workplaces and through word-of-mouth. Inclusion criteria for aphasia patients included (a) having a confirmed motor aphasia diagnosis, (b) 18 years of age or older, (c) ability to operate a computer mouse, and (d) right-hand dominance. Control group criteria were similar to those of the aphasia group, excluding medical diagnoses. Exclusion criteria for both groups were (a) comprehension impairments related to the brain lesion and (b) any history of neurological or psychiatric disorders that might interfere with this study. Informed consent was obtained from all participants, with the study protocol approved by the ethical committees of Sheba Medical Center (3710-16-SMC).

### 2.2. Instruments

#### 2.2.1. Confirmation of Aphasia Diagnosis

##### Bedside Western Aphasia Battery

The Bedside Western Aphasia Battery is a fundamental instrument for examining language deficits in individuals with aphasia. This extensive assessment tool encompasses multiple subtests that evaluate critical linguistic domains, including spontaneous verbal expression, comprehension of auditory input, repetition abilities, and word retrieval skills [64]. In this study, a revised bedside assessment was included, specifically developed for patients with acute conditions or severe impairments who could not complete the comprehensive assessment, maintaining the full evaluation in terms of overall severity ratings and individual subscale scores, despite its abbreviated format. The Bedside Western Aphasia Battery is used and was validated in diverse linguistic contexts, including Hebrew-speaking populations [65], and it was therefore employed in the current study.

#### 2.2.2. Assessment of General Intellectual Ability

##### Raven’s Colored Progressive Matrices Test (RCPMT)

A computerized version of the RCPMT [66] was used, based on the original Raven test [67], which was developed to assess fluid intelligence. This task included 36 items, which were systematically arranged into three sections of increasing complexity. The participants were shown geometric pattern matrices with an absent component in the lower right section. They needed to select the most suitable completion from six preferences to complete the pattern, with no time limitations during the assessment. The dependent variable was the total number of correctly completed items, ranging from 0 to 36.

#### 2.2.3. Assessment of WM

##### Tactual Span—One Hand

The Tactual Span task, which was originally designed for both hands [50], was modified and found valid and reliable in its one-hand version [56]. In this task, which was composed of two stages—forward and backward—a participant sat in front of a computer keyboard, placing four fingers of the left hand on four keys. To eliminate any visual cue, the participant wore eye covers. In the forward stage, the examiner, positioned opposite the participant, used the back of a pencil to touch each finger of the participant in a predetermined sequence. Each touch was held between the second and third knuckle of the finger for one second before moving to the next finger. The participant was then asked to press the corresponding keys in the exact order of the touches. The task began with sequences of two touches over three consecutive trials, and if at least one trial per sequence length was correct, an additional stimulus was added to the sequence until the participant failed all three trials at a particular length. The backward recall phase was identical, but the participant repeated the sequence in reverse order. The longest sequence accurately recalled in each stage was the dependent variable.

##### Visuospatial Span

The computerized Visuospatial Span task, which is based on the Corsi Block-Tapping test [39], was used to assess WM in the visuospatial modality. In this task, which was also composed of forward and backward stages, nine purple squares were displayed on the screen in a random arrangement. Each square changed color to yellow for one second in a predetermined sequence. For the forward recall phase, the participant was instructed to replicate the sequence by clicking on the squares in the same order, using a computer mouse. In the backward recall phase, the participant was asked to click on the squares in the reverse order of the original sequence. The task began with sequences of two squares, each presented in two trials, and if at least one trial was correctly recalled, an additional square was added to the next sequence length. This continued until the participant failed both trials at a given sequence length. The dependent variable was the longest sequence length correctly recalled in the forward and backward stages separately.

##### 1-Back Task

The computerized 1-Back task was used to evaluate WM in the visual domain [68,69]. The participant was seated facing a screen, viewing nine distinct facial images. The objective was to indicate when the displayed face matched the one shown previously. The assessment consisted of two blocks, containing 30 trials, with six target faces per block. Each facial stimulus remained visible for 1500 ms, with a 500 ms pause between presentations. The participant was asked to press the spacebar as quickly as possible when two identical consecutive faces were shown on the screen until the task ended. The dependent variable was the mean response time to target stimuli.

### 2.3. Procedure

Initial participant screening involved the recruitment of post-ischemic stroke motor aphasia patients through the rehabilitation department (*n* = 10) and referrals from private practice speech therapists (*n* = 4), with the Bedside Western Aphasia Battery used to confirm motor aphasia diagnosis and verify the absence of comprehension deficits. The healthy control group (*n* = 15) was recruited through workplace referrals and word-of-mouth recommendations. Participants were individually scheduled for experimental sessions, during which they provided written informed consent and completed demographic information forms. The experimental protocol comprised WM assessments across two modalities: tactile and visuospatial. Each participant underwent comprehensive testing. The entire experimental session was standardized, lasting approximately 45 min, ensuring consistent assessment conditions, with the participant sitting in a quiet room in the department or the participants’ homes, ensuring no interruptions. The tasks were counterbalanced by changing the place of each task in one position every time the battery was presented. Upon the completion of the experiment, participants were allowed to ask questions about the research and seek clarification about the study’s objectives and procedures.

### 2.4. Statistical Analysis

A preliminary analysis was conducted to compare the groups on demographic characteristics, employing a one-way Multivariate Analysis of Variance (MANOVA) for age, education, and intellectual ability, alongside a chi-square test for gender. Given the limited sample size, the Shapiro–Wilk test [70] was subsequently used to assess the normality of the dependent variables. Levene’s test was applied to evaluate the homogeneity of variance, and when that was not met, Welch’s test was used [71]. Then, a one-way MANOVA was conducted to examine differences between the aphasia and control groups across all WM measurements. Subsequently, for evaluating group classification, sensitivity, and specificity of Tactual Span scores relative to other WM measures, a receiver operator characteristic (ROC) analysis was performed. Data analysis was completed using SPSS version 29, with a statistical significance threshold determined at *p* < 0.05.

## 3. Results

The study sample included 29 right-handed individuals, who were divided into 14 patients (12 males) with left stroke-induced motor aphasia and 15 healthy individuals (13 males). All patients were diagnosed with aphasia by a certified speech therapist using the Bedside Western Aphasia Battery and also had a diagnosis of right hemiparesis.

Comparing the groups by age, education, and intellectual ability yielded nonsignificant findings (*F*(1, 29) = 2.1, *p* = 0.12). Additionally, a chi-square test for goodness of fit indicated no significant difference in gender distribution between the groups (χ^2^(1) = 0.006, *p* = 0.94; see Table 1 and Table 2).

The Shapiro–Wilk test for normality was applied to each group, yielding non-significant outcomes across all variables, which suggested normal distribution (see Appendix A). Additionally, Levene’s test for homogeneity of variances was conducted on all dependent variables. Significant variance was found for the Tactual Span forward (*F*(1, 27) = 13.97, *p* = 0.001) and Tactual Span backward (*F*(1, 27) = 42.13, *p* < 0.001). Consequently, parametric analyses were deemed suitable in all tasks except for the forward and backward Tactual Span.

**Figure 1:**
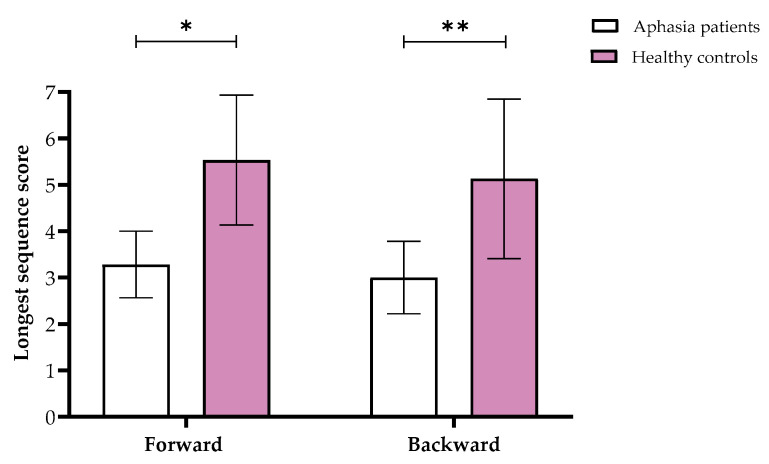
Means and standard deviations of the Tactual Span forward and backward longest sequence scores in the aphasia and healthy control groups. Note. * p < 0.05, ** *p* < 0.001.

Comparing the groups revealed a significant multivariate effect (*F*(1,29) = 8.34, *p* < 0.001, partial η^2^ = 0.64). Further univariate tests yielded significant differences in Tactual Span for both forward (Welch’s *F*(1,19.16) = 18.81, *p* < 0.001, partial η^2^ = 0.39) and backward (Welch’s *F*(1,17.36) = 22.54, *p* < 0.001, partial η^2^ = 0.44) stages (see Figure 1).

**Figure 2:**
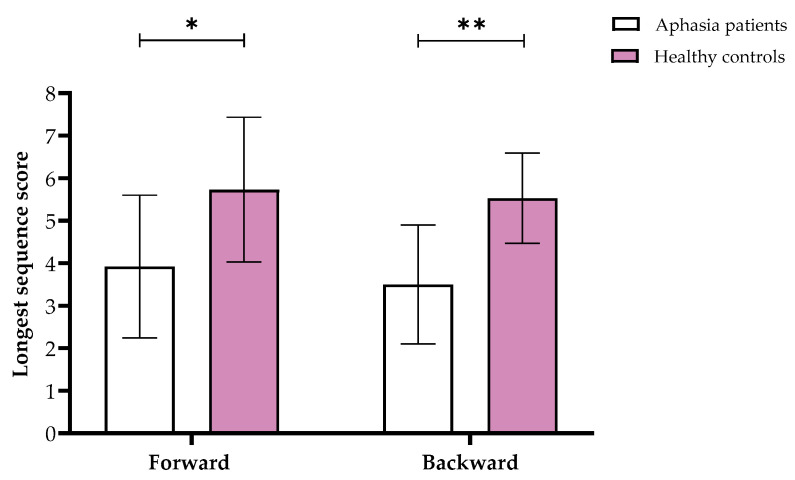
Means and standard deviations of the longest sequence scores for the Visuospatial Span forward and backward in the aphasia and healthy control groups. Note. * *p* < 0.05, ** *p* < 0.001.

Furthermore, we found significant differences in the Visuospatial Span in both forward (*F*(1,29) = 7.51, *p* = 0.011, partial η^2^ = 0.21) and backward (*F*(1,29) = 20.28, *p* < 0.001, partial η^2^ = 0.42) stages, showing that overall, aphasia patients performed worse than controls (see Figure 2). Similarly, the 1-Back task showed significant results *(F*(1,29) = 0.37, *p* = 0.002, partial η^2^ = 0.3), with the control group responding faster than the aphasia patients.

**Figure 3:**
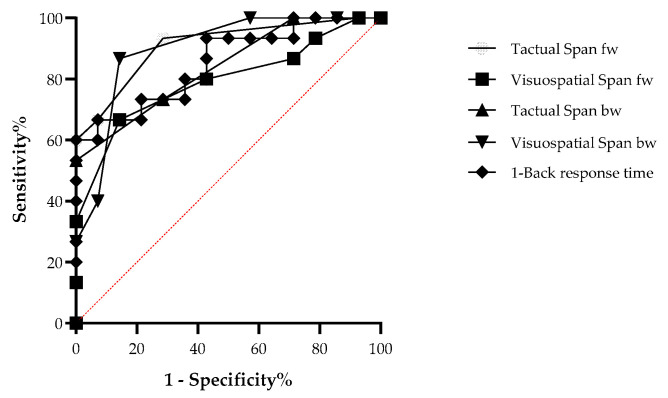
ROC curve comparing the aphasia patients and control groups in the working memory tasks.

Finally, ROC analysis was employed to assess discrimination strength using sensitivity and specificity measures. The results indicated that all WM variables significantly distinguished the aphasia group from the control group (see Table 3). All tasks, including Tactual Span forward and backward, showed good discrimination between patients and controls (see Figure 3).

## 4. Discussion

The current study objectives were to compare non-verbal WM functioning in tactile and visuospatial modalities among post-ischemic stroke motor aphasia patients and to validate the one-hand version of the Tactual Span for clinical use in this population. Despite the absence of intellectual differences between the groups, the findings showed worse performance of aphasia patients on the WM tasks. In addition, the Tactual Span was found to differentiate adequately between the groups in both the storage and manipulation components, while showing good specificity and sensitivity estimates. The other tasks were also found to distinguish between the groups.

The poor performance observed in aphasia patients across all WM tasks corroborates the findings of Kasselimis et al. [28], who identified deficits in both verbal and non-verbal WM among left-stroke patients with and without aphasia. This observation concurs with extensive research documenting non-verbal WM deficits in individuals with aphasia [3,7,28,32,72], suggesting that WM functions may not be strictly modality-dependent, although emerging evidence suggests that tactile WM tasks may tap into distinct neural networks [73,74,75,76].

Marinelli et al. [77] provided additional insights by emphasizing the intricate relationship between language and overall cognitive information processing. They emphasized that language supports various neurological mechanisms across different modalities. Consequently, damage to regions primarily responsible for verbal functions, such as the left hemisphere, may indirectly compromise non-verbal WM performance.

A complementary explanation for the poor performance observed in non-verbal WM focuses on the intrinsic nature of the left hemisphere. Despite its primary association with verbal cognitive functions, brain regions in the left hemisphere such as the fronto-parietal area, medio-frontal cortex, superior parietal lobule, and precuneus [31,78] also underpin non-verbal WM ability. In addition, it was also suggested that WM is supported by a complex neural network located in the left hemisphere, contributing to its holistic functionality [28,47,79]. This neurological perspective resonates with evolutionary theory, which posits that non-verbal functions engage both the right and left hemispheres. This dual hemispheric involvement stems from the relatively recent evolutionary development of language, in contrast to visual processing capabilities, which historically utilized both brain hemispheres before becoming more specialized [48]. Neuroimaging studies further substantiate this understanding, demonstrating that WM involves the maintenance of information through contributions from diverse non-verbal brain regions [49,80].

Fonseca et al. [1] conducted a comprehensive review of 38 studies on neuropsychological assessments utilizing non-verbal instruments and found that most studies have shown that patients with stroke-induced aphasia consistently received lower scores on non-verbal cognitive tests across various domains, compared to healthy individuals. However, no significant differences were identified when comparing these patients to individuals with left- or right-hemisphere damage without aphasia. Consequently, the authors concluded that some of the non-verbal impairments observed in aphasia patients are not secondary to language deficits but to brain dysfunction per se. Thus, the current study provides novel evidence demonstrating that the tactile modality of WM might be as compromised in individuals with aphasia as other modalities.

The primary aim of our study was to validate the Tactual Span task in aphasia patients. Considering that many neuropsychological assessment tools require verbal output [18], and the need for reliable tests for evaluating WM in individuals with aphasia [11,34], the Tactual Span emerges as a valuable clinical instrument. This is also important because the assessment of WM function has predominantly focused on visuospatial WM [3,31,72]. Therefore, the Tactual Span can provide further information from a different modality and can be especially useful for those patients with visual impairment. Thus, employing multiple tools to assess neurocognitive functioning provides a more comprehensive understanding of WM ability. Such a multidimensional approach enhances the accuracy of cognitive assessments, and concurs with the need for diverse evaluation methods in clinical practice [81,82]. Therefore, the Tactual Span task seems valid in measuring non-verbal WM in individuals with aphasia or patients with left-hemisphere brain damage. Its use by clinicians and assessment professionals can contribute to a more detailed understanding of non-verbal WM, enhancing the broader comprehension of cognitive impairments associated with aphasia.

This study has certain limitations, primarily concerning the small sample size, the time elapsed since the stroke, and the predominance of male participants. Overall, the results should be interpreted with caution, as the relatively broad AUC confidence intervals indicate uncertainty surrounding our estimates. Future research should address these constraints and further evaluate the utility of the Tactual Span in assessing WM across different aphasia types (e.g., anomic, conduction, or transcortical motor aphasia) and compare the performance of patients with left hemispheric stroke to those with right hemispheric stroke using this task. Such research would extend our understanding of the Tactual Span’s clinical applicability. Furthermore, studies could also explore the utility of the Tactual Span in cases of aphasia due to traumatic brain injury or brain tumors.

In conclusion, non-verbal WM appears to be impaired in patients with left-hemisphere strokes resulting in aphasia. The current pilot study has shown that the impact of stroke-induced motor aphasia on tactile modality is comparable to that of visuospatial modality. These results also support the effectiveness of using the Tactual Span as a clinical tool for WM assessment, which contributes to distinguishing between aphasia patients and healthy individuals. However, these conclusions should be interpreted with caution due to the small sample size. Researchers studying WM in aphasia patients should consider including both tactile and visuospatial tasks to capture the full spectrum of non-verbal cognitive deficits, as well as controlling for modality-specific effects. Clinicians working with post-stroke aphasia patients may benefit from using the Tactual Span, either as a part of their standard assessment battery or as a useful alternative when traditional methods are difficult to administer. Our findings suggest new research opportunities for studying WM function in individuals with aphasia and other types of brain injury, particularly concerning modality-specific WM evaluation.

## Figures and Tables

**Table 1:** Full demographic characteristics of participants.

Group	No.	Sex	Age	Education (Years)	Time After Stroke (Months)
Aphasia patients	1	Male	71	16	14
	2	Male	45	14	30
	3	Male	62	12	66
	4	Male	65	15	24
	5	Male	58	18	3
	6	Male	37	15	24
	7	Male	68	12	8
	8	Male	50	12	20
	9	Male	63	19	36
	10	Male	64	12	24
	11	Male	61	15	120
	12	Male	60	17	10
	13	Female	27	16	30
	14	Female	78	15	8
Controls	1	Male	40	17	--
	2	Male	49	15	--
	3	Male	67	18	--
	4	Male	47	12	--
	5	Male	57	16	--
	6	Male	44	16	--
	7	Male	65	20	--
	8	Male	35	17	--
	9	Male	33	17	--
	10	Male	37	20	--
	11	Male	42	17	--
	12	Male	53	15	--
	13	Male	47	14	--
	14	Female	74	16	--
	15	Female	46	16	--

**Table 2:** Means and standard deviations of demographic variables and working memory tasks among participants with aphasia and control groups.

Variable	Aphasia Patients (*n* = 14)	Control Group (*n* = 15)
Age (years)	57.78 (13.7)	49.06 (12.12)
Education (years)	14.78 (2.55)	16.40 (2.06)
Tactual Span forward	3.28 (0.72)	5.53 (1.41)
Visuospatial Span forward	3.92 (1.68)	5.73 (1.71)
Tactual Span backward	3.00 (0.78)	5.13 (1.73)
Visuospatial Span backward	3.50 (1.40)	5.53 (1.06)
1-back task (ms.)	0.78 (0.19)	0.55 (0.17)
RCMT (intellectual ability)	32.28 (10.99)	34.40 (1.63)
Time since stroke (months)	29.78 (30.37)	----

Note. RCPMT = Raven’s Colored Progressive Matrices Test.

**Table 3:** ROC curve estimates (with standard error for AUC measure in brackets) for aphasia patients and the control group.

Measure	Sensitivity (%)	Specificity (%)	AUC	95% CI	Cut-Off Score
TS fw	92.9	66.7	0.91 (0.05)	[0.80, 1.00]	4.5
VS fw	85.7	66.7	0.79 (0.08)	[0.62, 0.96]	5.5
TS bw	71.4	73.3	0.83 (0.07)	[0.69, 0.98]	3.5
VS bw	85.7	86.7	0.89 (0.06)	[0.77, 1.00]	4.50
1-Back task	85.7	73.3	0.81 (0.08)	[0.66, 0.97]	0.62

Note. AUC = Area Under the Curve; TS = Tactual Span; VS = Visuospatial Span; fw = forward; bw = backward; CI = confidence interval.

## Data Availability

All data, analysis code, and research materials are available from the corresponding author upon reasonable request.

## Appendix A

Shapiro–Wilk estimates of normality for all dependent variables among aphasia patients and controls.

**Variable**	**Aphasia Patients**		**Control Group**	
	Shapiro–Wilk	df	Shapiro–Wilk	df
Tactual Span forward	0.88	14	0.89	15
Visuospatial Span forward	0.93	14	0.94	15
Tactual Span backward	0.93	14	0.95	15
Visuospatial Span backward	0.91	14	0.87	15
1-Back task	0.97	14	0.88	15
Note: *df* = degrees of freedom.				
